# Factors associated with cholera in Kenya, 2008-2013

**DOI:** 10.11604/pamj.2017.28.101.12806

**Published:** 2017-10-03

**Authors:** Gretchen Cowman, Shikanga Otipo, Ian Njeru, Thomas Achia, Harsha Thirumurthy, Jamie Bartram, Jackson Kioko

**Affiliations:** 1 Department of Health Policy and Management, University of North Carolina, Chapel Hill, North Carolina, United States of America; 2 Disease Surveillance and Response Unit, Ministry of Health, Nairobi, Kenya; 3 School of Public Health, University of Witwatersrand, Johannesburg, South Africa; 4 The Water Institute, Department of Environmental Sciences and Engineering, University of North Carolina, Chapel Hill, North Carolina, United States of America; 5 Department of Preventive and Promotive Health, Ministry of Health, Nairobi, Kenya

**Keywords:** Cholera, disease surveillance, Kenya, open defecation, sanitation, water

## Abstract

**Introduction:**

Kenya experienced widespread cholera outbreaks in 1997-1999 and 2007-2010. The re-emergence of cholera in Kenya in 2015 indicates that cholera remains a public health threat. Understanding past outbreaks is important for preventing future outbreaks. This study investigated the relationship between cholera occurrence in Kenya and various environmental and demographic factors related to water, sanitation, socio-economic status, education, urbanization and availability of health facilities during the time period 2008-2013.

**Methods:**

The primary outcome analyzed was the number of cholera cases at the district level, obtained from the Kenya Ministry of Health's national cholera surveillance records. Values of independent variables were obtained from the 2009 Kenya Population and Housing Census and other national surveys. The data were analyzed using a zero-inflated negative binomial regression model.

**Results:**

Multivariate analysis indicated that the risk of cholera was associated with open defecation, use of unimproved water sources, poverty headcount ratio and the number of health facilities per 100,000 population (p < 0.05). No statistically significant association was found between cholera occurrence and education, percentage of population living in urban areas or population density.

**Conclusion:**

The Sustainable Development Goals and Kenya's blueprint for development, *Kenya Vision 2030*, call for access to sanitation facilities and clean water for all by 2030. Kenya has made important economic strides in recent years but continues to be affected by diseases like cholera that are associated with low socio-economic status. Further expansion of access to sanitation facilities and clean water is necessary for preventing cholera in Kenya.

## Introduction

Cholera is an epidemic-prone diarrheal disease of global significance. The disease is endemic in Kenya and the country experienced large, widespread outbreaks in 1997-1999 and in 2007-2010 [1]. Between 2011 and 2014 there was a dramatic decrease in the number of reported cholera cases in Kenya. However, in 2015 cholera re-emerged in Kenya and it remains a serious threat to public health. A number of epidemiological investigations have been published on cholera outbreaks in Kenya [1-9]. Identified risk factors include: water, sanitation and hygiene factors; childhood age; lack of knowledge about cholera; proximity to a large body of water; and limited availability of healthcare facilities, oral rehydration solution and intravenous fluids. Poverty, living in a refugee camp and rainfall/flooding have also been implicated as risk factors for cholera in studies outside of Kenya [10-12]. Cholera surveillance and response in Kenya is guided by the Integrated Disease Surveillance and Response (IDSR) strategy. The IDSR technical guidelines require case-based reporting of cholera within 24 hours of detection, and health facilities submit weekly disease summary reports that include data on cholera [13]. Surveillance data on cholera and other priority diseases is compiled at national level by the Ministry of Health and disseminated in a weekly epidemiological bulletin. Safe drinking water and basic sanitation are essential to preventing diarrheal diseases like cholera. A large portion of the population in Kenya lacks access to an improved water source or an improved sanitation facility. Between 1990 and 2015, the percentage of the overall population that used improved drinking water sources increased from 43% to 63% [14]. In urban areas, however, the percentage of population using improved water sources actually decreased from 92% to 82% during the same period. Only modest progress has been seen in Kenya with respect to sanitation. The percentage of the population using improved sanitation facilities increased from 25% in 1990 to 30% in 2015. The percentage of the population practicing open defecation decreased from 19% in 1990 to 12% in 2015 [14]. One sanitation strategy that is being implemented at large scale in Kenya is Community-Led Total Sanitation (CLTS). In 2010 CLTS was pilot tested in 6 districts in Western Kenya [15] and subsequently adopted by the Ministry of Health as part of the national strategy to eliminate open defecation. Despite the vast body of knowledge on cholera, prevention and control of this disease remains a challenge in Kenya. A better understanding is needed of the role that various risk factors play in cholera outbreaks in Kenya. This study investigated the association between cholera occurrence in Kenya and environmental and demographic factors related to water, sanitation, socio-economic status, education, urbanization and availability of health facilities over the time period 2008-2013. This study also seeks to contribute to the knowledge base necessary for developing effective policies and strategies that can be successfully implemented in limited resource settings to prevent cholera.

## Methods

The investigation was based on a cross-district analysis of cholera occurrence. The primary outcome examined was the number of cholera cases at the district level. The time period of the study, 2008-2013, represents three years of widespread cholera outbreaks in Kenya and three years of few, isolated outbreaks.

**Data sources**: The primary data source for the number of cholera cases at the district level was the national cholera surveillance data collected and maintained by the Disease Surveillance and Response Unit of the Kenya Ministry of Health. These data were routinely collected through a district-based reporting system in accordance with Kenya's Integrated Disease Surveillance and Response (IDSR) strategy. The data were available in the form of a spreadsheet of aggregate numbers of cholera cases by district and year, de-identified line lists and the Ministry of Health's Weekly Epidemiological Bulletin. There was a gap in the data for 2011 and the number of reported cholera cases for this year was obtained from the World Health Organization's Weekly Epidemiological Record [16]. Supplemental information on locations of cholera occurrence was obtained from outbreak reports posted by the Program for Monitoring Emerging Diseases (ProMED-mail) [17]. Population data were obtained from the 2009 Kenya Population and Housing Census [18]. Data on environmental and demographic factors at the district level were extracted from published survey reports and from a 10% micro-data set of the 2009 census available from the Kenya National Bureau of Statistics. The variables included in this study were chosen based on findings in the literature about risk factors for cholera. Definitions and data sources for these factors are provided in Table 1.

**Table 1:** Environmental and demographic factors with their definitions and data sources

Factor	Definition	Data source
Percentage of households practicing open defecation	Number of households practicing open defecation divided by the total number of households, expressed as a percentage. For the purpose of this study open defecation includes the following modes of human waste disposal from the 2009 census: bush, bucket, and other.	2009 Kenya Population and Housing Census
Percentage of households using an improved water source	Number of households with access to an improved water source divided by the total number of households, expressed as a percentage. Improved water sources include the following categories from the 2009 census: protected spring, protected well, borehole, piped into dwelling, piped, rain water collection; does not include pond, dam, lake, stream/river, unprotected spring, unprotected well, jabia^+^, water vendor, or other.	2009 Kenya Population and Housing Census, 10% micro-data set
Poverty headcount ratio	Percentage of persons living below the poverty line, calculated as the number of persons living below the poverty line divided by the total population.	2005/2006 Kenya Integrated Household Budget Survey data as presented in “Exploring Kenya’s Inequality” report, 2013
Percentage of population with at least some secondary education	Number of people with at least some secondary or higher level education divided by the total population, expressed as a percentage. From the 2009 census the following categories of highest level of education reached were included: secondary, tertiary, university, and youth polytechnic. The following categories were not included: never attended, pre-primary, primary, basic literacy, and madrassa.	2009 Kenya Population and Housing Census
Percentage of population living in urban areas	Population living in urban centers (settlements with population estimated at 2,000 or higher during the 2009 census) divided by total population, expressed as a percentage.	2009 Kenya Population and Housing Census
Population density	Number of people per unit area. In the 2009 census, population density is expressed as number of people per square kilometer.	2009 Kenya Population and Housing Census
Number of health facilities per 100,000 population	Number of healthcare facilities in a district divided by district population and expressed per 100,000 people. Healthcare facilities include public, non-governmental, faith-based, and private facilities. Data were derived from the Ministry of Health’s Master Facility List. The following categories of facilities were not included: HIV counseling and testing center, nursing home, regional blood transfusion center, training institution, dental clinic, laboratory, radiology unit, health project, and facilities labeled as “not operational” or “pending opening.”	Ministry of Health Master Facility List, http://www.ehealth.or.keaccessed 23 Oct 2013

+ “jabia” is a term used in Kenya to describe a traditional rainwater storage system

**Data analysis**: The unit of analysis was the district and the investigation was based on the geographic boundaries of the 158 administrative districts in the 2009 census. The analysis began with a review of the completeness of cholera surveillance data and consistency among available data sources for cholera case counts. The Ministry of Health spreadsheet of aggregate case counts and line lists were used as the primary data sources and secondary data sources were used to estimate missing values and to check consistency in terms of presence or absence of cholera in a district for a given year. Cholera surveillance data could not be fully resolved among the 158 districts in the 2009 census. The number of administrative districts in the country has varied over time from 47 districts in 1992 to 254 districts by the end of 2009. As a result, cholera data for some of the 158 districts was combined in some years. For example, cholera cases for Kisumu East and Kisumu West districts were reported as a single district, Kisumu, in the primary data source in 2008 and 2009. A total of 35 districts were affected in this way. Following aggregation of districts based on data availability, a total of 137 district units were used in the regression analysis.

District level cholera data were aggregated over the 6-year period 2008-2013. Point estimates for environmental and demographic factors were obtained from the data sources identified in Table 1. Statistical analysis was performed in STATA (StataCorp, version 14.1). Potential co-linearity between independent variables was investigated using Pearson's correlation coefficient. Association between number of cholera cases and the independent variables was investigated by univariate and multivariate analysis using a zero-inflated negative binomial (ZINB) regression model. Negative binomial regression was selected since the outcome (number of cholera cases) is count data, a preliminary review of scatterplots suggested that potential associations may not be linear and the cholera data were over-dispersed. An adjustment for zero inflation was included since 80 of 137 districts did not report any cases of cholera between 2008 and 2013. The fact that a district did not report any cases of cholera may reflect true absence of cholera, or it may be the result of the district not detecting or reporting cases of cholera. In addition to adjusting for zero inflation, the model incorporated an offset of the logarithm of the 2009 district population to adjust for population size. Independent variables measured as percentages were converted to categorical variables and divided into terciles, yielding low, mid and high categories for comparison. Population density and number of health facilities per 100,000 people were analyzed as continuous variables. A multivariate model was built by forward selection, beginning with the most significant variable and sequentially adding variables that improved the model fit. Models were compared by a likelihood ratios test to determine the best fitting model.

**Ethics review**: The Kenya Medical Research Institute Ethics Review Committee granted ethical approval for this study (Non-SSC 438), and the University of North Carolina Office of Human Research Ethics granted an Institutional Review Board exemption (study no. 13-3399).

## Results

Between 2008 and 2013 an estimated 17,917 cholera cases were reported nationally. Cumulative cholera incidence ranged from a low of 0 cases per 100,000 in 80 districts to a high of 884 cases per 100,000 in three combined districts in northern Kenya. Between 2008 and 2010 cholera was widespread in Kenya affecting populations in a variety of geographic landscapes and climatic zones, including arid and semi-arid regions of the country, low lying areas along the Indian Ocean coast with a monsoon type climate and areas with a tropical, humid climate bordering Lake Victoria. Cholera affected both rural and urban areas. Some of the least densely populated areas of northern Kenya reported large outbreaks while Kenya's largest cities, including Nairobi, Mombasa and Kisumu, also reported outbreaks. Between 2011 and 2013 cholera outbreaks were limited to Dadaab Refugee Camp and surrounding areas in northeastern Kenya. Conditions related to the environmental and demographic factors of interest varied considerably across the country. The range of values of these factors is summarized in Table 2 for the 137 districts that were included in regression analyses. Open defecation, education and poverty were highly correlated with each other, with the absolute values of Pearson's correlation coefficients ranging from 0.69 to 0.77 (p < 0.0001). Districts with a higher percentage of households practicing open defecation tended to have a higher poverty headcount ratio and a lower percentage of population with at least some secondary education.

**Table 2:** Range of values of environmental and demographic factors, Kenya, 2008-2013, n=137 districts

Factor	Range of values
% of households practicing open defecation	0.1-94.6%
% of households using an improved water source	10.8-84.1%
Poverty headcount ratio	18.3-87.5%
% of population with at least some secondary education	2.1-46.1%
% of population living in urban areas	0.0-100.0%
Population density	3-4,515 people per square kilometer
# of health facilities per 100,000 population	6.0-76.5

Univariate analysis using ZINB regression models suggested a statistically significant association (p < 0.05) between cholera occurrence and each of the factors investigated except for percentage of population living in urban areas. In Table 3, relative risks are presented for each of the factors considered. For open defecation, water source, poverty, education and urbanization, the relative risk for the mid (33^rd^ to 66^th^ percentile) and high (66^th^ percentile and above) categories is presented relative to the lowest category. The best fitting multivariate model contained open defecation, water source, poverty and number of health facilities as significant factors (p < 0.05). There was a statistically significant higher risk of cholera in districts with mid to high levels of open defecation compared to districts in the lowest category (ARR 3.62, 95% CI 1.56-8.39 and ARR 3.84, 95% CI 1.49-9.85, respectively). The risk of cholera was significantly higher in districts in the high poverty category compared to the low poverty category (ARR 3.29, 95% CI 1.21-8.94), but no significant difference in risk was seen between the mid and low poverty categories (ARR 0.82, 95% CI 0.32-2.10). Cholera risk was lower in districts with a high level of use of improved water sources compared to districts with a low level of use (ARR 0.43, 95% CI 0.21-0.89), but no significant difference was observed between the mid and low categories (ARR 0.96, 95% CI 0.48-1.91). Districts with a higher number of health facilities per 100,000 tended to have a higher number of reported cholera cases (ARR 1.06, 95% CI 1.02-1.10). The percentage of the population living in urban areas was the only factor that was a statistically significant predictor of zero inflation on its own and was therefore used in the zero inflation portion of the model. This factor had a negative relationship with zero inflation, suggesting that districts with a lower percentage of population living in urban areas had a higher probability of excess zeros (that is, not reporting cases).

**Table 3:** Results of zero-inflated negative binomial regression, Kenya, 2008-2013, n=137 districts

Factor	RR (95% CI)	ARR (95% CI)
**% of households practicing open defecation**		
low	1.00 (Reference)	1.00 (Reference)
mid	3.82^++^ (1.39-10.49)	3.62^++^(1.56-8.39)
high	8.85^+++^(3.39-23.15)	3.84^++^(1.49-9.85)
**% of households using an improved water source**		
low	1.00 (Reference)	1.00 (Reference)
mid	0.80 (0.36-1.81)	0.96 (0.48-1.91)
high	0.26^++^(0.11-0.61)	0.43^+^(0.21-0.89)
**Poverty headcount ratio**		
low	1.00 (Reference)	1.00 (Reference)
mid	0.91 (0.39-2.11)	0.82 (0.32-2.10)
high	4.11^+++^(1.86-9.08)	3.29^+^(1.21-8.94)
**% of population with at least some secondary education**		
low	1.00 (Reference)	
mid	0.32^++^(0.15-0.69)	
high	0.17^+++^(0.08-0.39)	
**% of population living in urban areas**		
low	1.00 (Reference)	
mid	1.73 (0.68-4.40)	
high	0.97 (0.39-2.44)	
Population density	0.9995^++^(0.9992-0.9998)	
# of health facilities per 100,000	1.05^+^(1.00-1.10)	1.06^++^(1.02-1.10)

+ p<0.05

++ p<0.01

+++ p<0.001 RR = relative risk, ARR = adjusted relative risk

## Discussion

The results of this study suggest that districts in Kenya with a higher prevalence of open defecation are at higher risk of cholera. To the best of our knowledge, this is the first study to show an association between cholera occurrence and open defecation. Other studies have investigated the relationship between cholera and other measures of sanitation with mixed results. In a multi-country study, Leidner et al found a negative correlation between cholera incidence and access to improved sanitation facilities [19]. In an analysis from Kenya covering the period 1999-2009, Stoltzfus et al did not detect an association between cholera incidence and the percentage of population using unsafe sanitation facilities (defined as anything other than flush toilet, ventilated improved pit latrine or pit latrine) [9]. The authors noted, however, that lack of variability in some of the independent variables across 69 districts and lack of statistical power may have limited the ability of the study to detect associations among some of the variables. Nearly one billion people worldwide practice open defecation [14] and this has been recognized as a global challenge. In 2013 the United Nations launched a call to action on sanitation with the aim of eliminating open defecation globally by 2025 [14]. This is supported by the Sustainable Development Goals, which also call for an end to open defecation [20]. The results of this study suggest that reducing open defecation may be essential to preventing cholera in Kenya.

This study also found an inverse relationship between cholera occurrence and use of an improved water source, suggesting a protective effect of improved water sources. This is similar to a finding by Leidner et al of a statistically significant relationship between cholera incidence and access to improved water sources [19]. An earlier study in Kenya, however, found no significant relationship, in multivariate analysis, between cholera incidence and the percentage of population without a piped water supply, which is one type of improved water source [9]. Access to improved water sources like piped water does not necessarily guarantee a clean water supply. Ensuring that improved water sources supply clean water is an important consideration. Although Kenya achieved an overall increase in percentage of population with access to improved water sources from 43% in 1990 to 63% in 2015, it must be noted that access in urban areas actually declined during this period. This is likely due to population growth in urban slums without adequate expansion of drinking water infrastructure. As efforts continue to expand access to clean water in rural areas, cholera outbreaks in Kenya's cities remind us that expanded access to clean water is needed in urban areas as well. In 2007 the Government of Kenya launched a development blueprint, *Kenya Vision 2030*, aimed at transforming Kenya into a 'middle-income country providing a high quality of life to all its citizens by the year 2030' [21]. This blueprint includes ensuring access to improved water and sanitation for all by 2030. This aligns with the Sustainable Development Goal for water and sanitation that aims to achieve access to safe and affordable drinking water as well as adequate and equitable sanitation and hygiene for all by 2030 [20]. The results of this study suggest that further achievements in Kenya with respect to water and sanitation are necessary for controlling cholera.

The results of this study suggest that cholera burden is also associated with poverty. Other studies have found this association as well [11, 12] and cholera is often referred to as a disease of poverty. This study did not attempt to investigate mechanisms by which poverty may influence cholera risk, but it was noted that poverty and open defecation are highly correlated in Kenya. An unexpected finding of this study was the higher adjusted number of cholera cases in districts with a higher number of health facilities per 100,000 people since it was anticipated that access to health facilities would reduce the spread of cholera. Possible explanations for this finding include a higher probability of detecting and reporting cholera cases in districts with a greater number of health facilities per population, cholera transmission may be occurring within health facilities, or there may be limitations in the data. This study did not find an association between cholera occurrence and population density or percentage of population living in urban areas. This supports similar results related to population density from a previous study in Kenya [9]. Observed patterns of cholera occurrence in Kenya indicate that cholera affects both urban and rural areas, including the most densely populated and least densely populated regions. This study also did not find an association between cholera occurrence and education. It is difficult to draw conclusions from this finding, however, given the high degree of co-linearity that was observed between education, open defecation and poverty. This study is subject to several limitations. One is potential regional variations in surveillance capacity that could affect the quality of cholera surveillance data. This study attempted to mitigate potential variability in reporting across districts by using cholera data over several years. There is some uncertainty in population figures used in this study. The 2009 census noted anomalies in population figures for several districts in northwestern and northeastern Kenya. There is an inherent limitation in the fact that the relational nature of this study does not prove or disprove causality between the independent and dependent variables. More detailed case-control studies or individual case investigations would be necessary to establish causes of cholera outbreaks. There is also an inherent limitation in the unit of analysis for this study. Data were analyzed at district level, yet it is recognized that differences in cholera risk exist at much smaller geographic units.

## Conclusion

Despite economic progress and improvements in a number of health and development-related indicators, cholera remains a significant public health challenge in Kenya. Kenya's Ministry of Health has collected routine cholera surveillance data over the years. These data are an important resource for understanding cholera occurrence in Kenya and can serve as an evidence base for developing effective strategies for preventing cholera. This study suggests that open defecation, lack of access to improved water sources and poverty are important contributors to cholera outbreaks in Kenya. On average 12% of Kenya's population practices open defecation and this figure is as high as 95% in some areas. Improvements in sanitation have been limited in the past 25 years and there is need to intensify efforts to eliminate open defecation and promote sanitation facilities that ensure safe disposal of human waste. Despite some progress with respect to drinking water, 37% of Kenyans still lack access to an improved water source and the problem is worsening in urban areas. Interventions that achieve very basic levels of environmental health by eliminating open defecation and expanding availability of improved water sources may be effective in preventing cholera. As Kenya sets its sights on achieving middle-income status, the country should strengthen efforts to ensure that sanitation facilities and clean water are available to all.

### What is known about this topic

- *Vibrio cholera* is a waterborne enteric pathogen spread through the fecal-oral route of transmission;
- Cholera tends to occur in poor communities where a large proportion of the population lacks access to improved water sources and/or improved sanitation;
- Kenya has experienced large cholera outbreaks in the past and the disease continues to be a public health threat in Kenya.

### What this study adds

- This study provides evidence for an association between cholera occurrence and open defecation. This is distinct from other studies that investigated the association between cholera occurrence and the broader category of unimproved sanitation;
- This study demonstrates the usefulness of national surveillance data collected through the IDSR strategy to analyze a public health problem and to potentially recommend public health actions;
- This study suggests that despite the recent economic progress achieved by Kenya, expanded access to improved water sources and sanitation is still needed to prevent cholera outbreaks in the future.

## Competing interests

The authors declare no competing interests.

## References

[1] Mutonga D, Langat D, Mwangi D, Tonui J, Njeru M, Abade A (2013). National surveillance data on the epidemiology of cholera in Kenya, 1997-2010. J Infect Dis..

[2] Shapiro RL, Otieno MR, Adcock PM, Phillips-Howard PA, Hawley WA, Kumar L (1999). Transmission of epidemic Vibrio cholerae O1 in rural western Kenya associated with drinking water from Lake Victoria: an environmental reservoir for cholera. Am J Trop Med Hyg..

[3] Mugoya I, Kariuki S, Galgalo T, Njuguna C, Omollo J, Njoroge J (2008). Rapid spread of Vibrio cholerae O1 throughout Kenya, 2005. Am J Trop Med Hyg..

[4] Mohamed AA, Oundo J, Kariuki SM, Boga HI, Sharif SK, Akhwale W (2012). Molecular epidemiology of geographically dispersed Vibrio cholerae, Kenya, January 2009-May 2010. Emerg Infect Dis..

[5] Mahamud AS, Ahmed JA, Nyoka R, Auko E, Kahi V, Ndirangu J (2012). Epidemic cholera in Kakuma Refugee Camp, Kenya, 2009: the importance of sanitation and soap. J Infect Dev Ctries..

[6] Loharikar A, Briere E, Ope M, Langat D, Njeru I, Gathigi L (2013). A national cholera epidemic with high case fatality rates-Kenya 2009. J Infect Dis..

[7] Onyango D, Karambu S, Abade A, Amwayi S, Omolo J (2013). High case fatality cholera outbreak in western Kenya, August 2010. Pan Afr Med J..

[8] Shikanga OT, Mutonga D, Abade M, Amwayi S, Ope M, Limo H (2009). High mortality in a cholera outbreak in western Kenya after post-election violence in 200. Am J Trop Med Hyg..

[9] Stoltzfus (2014). Interaction between climatic, environmental and demographic factors on cholera outbreaks in Kenya. Infect Dis Poverty..

[10] Griffith DC, Kelly-Hope LA, Miller MA (2006). Review of reported cholera outbreaks worldwide, 1995-2005. Am J Trop Med Hyg..

[11] Root ED, Rodd J, Yunus M, Emch M (2013). The role of socioeconomic status in longitudinal trends of cholera in Matlab, Bangladesh, 1993-2007. PLoS Negl Trop Dis..

[12] Talavera A, Perez EM (2009). Is cholera disease associated with poverty. J Infect Dev Ctries..

[13] Ministry of Public Health and Sanitation, Republic of Kenya (2012). Technical Guidelines for Integrated Disease Surveillance and Response in Kenya.

[14] UNICEF and World Health Organization (2015). Progress on sanitation and drinking water-2015 update and MDG assessment.

[15] Ministry of Public Health and Sanitation, Republic of Kenya (2012). Accelerating Access to Rural Sanitation in Kenya.

[16] World Health Organization (2012). Cholera, 2011. Wkly Epidemiol Rec..

[17] ProMED-mail (2014). International Society for Infectious Diseases. ProMED-mail.

[18] Kenya National Bureau of Statistics (2010). 2009 Kenya Population and Housing Census. Nairobi: Ministry of Planning, National Development and Vision 2030.

[19] Leidner AJ, Adusumilli NC (2013). Estimating effects of improved drinking water and sanitation on cholera. Journal of Water and Health..

[20] United Nations (2016). Sustainable Development Goals, Goal 6: ensure access to water and sanitation for all.

[21] Government of the Republic of Kenya (2007). Kenya Vision 2030: The popular version.

